# The effects of local variations in conditions on carbon storage and release in the continental mantle

**DOI:** 10.1093/nsr/nwae098

**Published:** 2024-03-18

**Authors:** Stephen F Foley, Chunfei Chen, Dorrit E Jacob

**Affiliations:** School of Natural Sciences, Macquarie University, North Ryde 2109, New South Wales, Australia; Research School of Earth Sciences, Australian National University, Canberra, AT 2601, Australia; School of Natural Sciences, Macquarie University, North Ryde 2109, New South Wales, Australia; State Key Laboratory of Geological Processes and Mineral Resources, School of Earth Sciences, China University of Geosciences, Wuhan 430074, China; Research School of Earth Sciences, Australian National University, Canberra, AT 2601, Australia

**Keywords:** lithospheric mantle, carbonate melts, deep carbon cycle, craton destruction, continental rifts

## Abstract

Recent advances indicate that the amount of carbon released by gradual degassing from the mantle needs to be revised upwards, whereas the carbon supplied by plumes may have been overestimated in the past. Variations in rock types and oxidation state may be very local and exert strong influences on carbon storage and release mechanisms. Deep subduction may be prevented by diapirism in thick sedimentary packages, whereas carbonates in thinner sequences may be subducted. Carbonates stored in the mantle transition zone will melt when they heat up, recognized by coupled stable isotope systems (e.g. Mg, Zn, Ca). There is no single ‘mantle oxygen fugacity’, particularly in the thermal boundary layer (TBL) and lowermost lithosphere, where very local mixtures of rock types coexist. Carbonate-rich melts from either subduction or melting of the uppermost asthenosphere trap carbon by redox freezing or as carbonate-rich dykes in this zone. Deeply derived, reduced melts may form further diamond reservoirs, recognized as polycrystalline diamonds associated with websteritic silicate minerals. Carbon is released by either edge-driven convection, which tears sections of the TBL and lower lithosphere down so that they melt by a mixture of heating and oxidation, or by lateral advection of solids beneath rifts. Both mechanisms operate at steps in lithosphere thickness and result in carbonate-rich melts, explaining the spatial association of craton edges and carbonate-rich magmatism. High-pressure experiments on individual rock types, and increasingly on reactions between rocks and melts, are fine-tuning our understanding of processes and turning up unexpected results that are not seen in studies of single rocks. Future research should concentrate on elucidating local variations and integrating these with the interpretation of geophysical signals. Global concepts such as average sediment compositions and a uniform mantle oxidation state are not appropriate for small-scale processes; an increased focus on local variations will help to refine carbon budget models.

## Teaser

Carbon is moved around in the Earth’s mantle in melts and fluids. This review shows that accurate estimations of the amount of carbon moved must consider local variations in temperature, oxidation state, and rock types deep in the mantle.

## INTRODUCTION

Improved quantification of the carbon contents of various reservoirs in the deep carbon cycle depends on progress in our understanding of the processes underlying the melting, devolatilization and transport of carbon in the deep Earth. Several reviews of inputs, outputs and reservoirs between 2009 and 2022 focussed on the deep carbon cycle as a whole, or on parts of it, including subduction or the continental lithosphere [1–5]. This review provides an update on developments over the last 6 years, especially regarding processes deep in subduction zones and at the base of the continental lithosphere. We concentrate on petrological and geochemical processes and mechanisms in the upper mantle rather than attempting to fix specific values on them. Revisions have also been made to CO_2_ outputs [6] and to volatile components other than carbon that are transported by similar melts and fluids, which are beyond the scope of this review. We restrict ourselves here to carbon; readers should consult other reviews for other volatile components such as H_2_O, sulphur, nitrogen, fluorine, chlorine and phosphorus [7–9].

Much of the focus here is to emphasize variations in space and, to a lesser extent, in time. Early budgets tended to be global, whereas more studies in the last few years have spotlighted local or regional variations. Looking at subduction, Bekaert *et al.* [10] noted, ‘We emphasize that the relative thickness, composition and ultimate contribution … to slab volatile inventories may vary greatly, and there is no single representative sample of subducting crust and oceanic lithosphere’. Similarly, Arzilli *et al.* [11] state that ‘quantifying the depth and amount of CO_2_ released from different carbonate-bearing lithologies during subduction is fundamental to understanding whether CO_2_ is recycled through arc volcanism or buried in the mantle’. High-pressure experiments and geodynamic models have increasingly considered the melting and rheology of individual sedimentary rock types to supplement those on average subducted sediment. These have shown some surprising results: examples are diapirism of thick subducting sediments in the solid state [12] and the generation of silicate melts from limestones [13].

**Figure 1.**
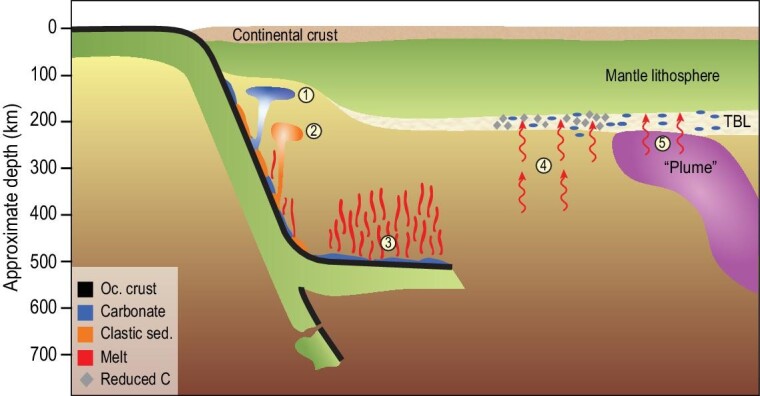
Summary of carbon transport processes in subduction and continental lithosphere. During subduction, thick carbonate sediments will rise diapirically [13], preventing deep subduction (1) as do clastic sediments in hot, wet conditions [120] (2). Thin carbonate sequences will be deeply subducted and may melt when stagnant slabs heat up in the mantle transition zone (3). Under the continents, the amount of gradual carbon degassing into the lithosphere (4) has been revised upwards, whereas plume input (5) may have been overestimated. Most carbon is trapped in the lithosphere in reduced form from melts, with carbonate-rich dykes forming only where the volume of introduced melt is high.

The storage and release of carbon in the continental lithosphere depend on several mechanisms of enrichment [4], each of which has been revised recently, and on estimates of the volume and the mechanisms of erosion of the lithosphere, particularly during rifting. Figure 1 summarizes the revision agenda for the continental lithosphere, concentrating on the fundamental processes that underpin estimates of carbon flux, which are needed to refine the quantification of the deep carbon cycle. Relative to the 2017 summary [4], recent work suggests that the amount of carbon added to the lithosphere by gradual enrichment should be revised upwards, plume inputs downwards, and that the processes of recycling of subducted crust and sediments must be considered in detail. We consider recent experimental evidence for the mobility of carbon in melts close to the lithosphere–asthenosphere boundary and for the behaviour of different types of subducted material. Here too, we emphasize spatial and temporal variations that will need to be considered in order to improve quantification of carbon reservoir sizes, budgets and fluxes.

## SOURCES OF CARBON

The original carbon content of the Archean cratonic lithosphere was estimated as the difference between volcanic output and the estimated subduction carbon flux, amounting to <90 ppm [4], assuming that this lithosphere was formed at subduction zones. Michaut *et al.* [14] note that long-term preservation of a cold and strong lithospheric root requires a cold initial temperature, which is best served by a subduction environment amongst the diverse mechanisms suggested for Archean craton formation [15]. A recent update of volcanic CO_2_ emissions revised estimates downwards [6], which would imply a decrease in the difference between subduction and emission and so the storage of less carbon in the lithosphere before modifications took place. However, the diffusive loss of CO_2_ along faults has been shown to dominate degassing in continental rift zones but is an unknown quantity in island arcs, potentially resulting in a large amount of carbon to account for [6,16]. There is evidence from carbon isotopes that a significant proportion of degassing CO_2_ is of crustal origin [17], which may be related to the reactivation of downthrust carbonate sediments in collision zones that are never truly subducted [18].

It has been suggested that some of the imbalance between subduction and emission may be attributed to consumption by microbes—a possible sink on the modern Earth that would not apply to the early Earth. A strongly geochemically depleted composition is accepted for the Archean continental lithosphere, which is consistent with a low carbon content, bearing in mind the geochemical incompatibility of CO_2_. We will return to the alternative of carbonate storage in the forearc [6] in a later section.

Carbon enrichment of the lithosphere by gradual degassing of the mantle over time was estimated as 100 ppm [4], as an intermediate round number within the range of 72–184 ppm estimated at the time [1,19,20]. Several recent papers using different methods agree that this value should be higher: Aiuppa *et al.* [21] analysed melt inclusions, arriving at an average of 352 ppm, and emphasizing the heterogeneous distribution of carbon in these inclusions (range 117–669 ppm). Shimizu *et al.* [22] used CO_2_/Ba ratios to arrive at a value of 391 ppm C in the mantle source of Pacific E-MORB and suggested that one possible source of carbon might be the foundering of continental lithosphere, which would add another type of recycling to the mix. For Iceland, a new value of 368 ppm [23] is higher than earlier estimates, although their deep mantle value of 1350 ppm may reflect the input of recycled carbon from the plume. Together, these more recent literature studies would appear to imply that ≈370 ppm is a more realistic average value. Diamonds are the most direct source of information about reduced carbon in the lithosphere, but their simple composition provides few clues as to their origin. The carbon isotope record is essentially unchanged over the last 3 Ga [24], indicating that assuming linear accumulation of carbon is probably justified. However, in estimating the time-integrated storage of carbon in cratonic lithosphere, it is now recognized that many areas have thick lithosphere of Proterozoic age than Archean, for example, large expanses of Australia [25]. This counteracts the time-integrated effect on carbon accumulation in the lithosphere by reducing the average age of the lithosphere (2.81 Ga) used in [4].

## REASSESSING PLUME INPUT

The contribution of carbon to the continental lithosphere from plumes, with the carbon derived either from the deep mantle or from lithological components of recycled oceanic crust, is very poorly known. The frequency of passage of plumes beneath any continent every 600–700 million years [4] is probably fairly accurate, whereas the carbon content of plumes will depend on the amount and type of recycled material in the plume, as well as the residence time and depth and extent of remixing into the mantle. In concluding that plumes contributed more carbon to the lithosphere than gradual degassing of the deeper mantle, Foley and Fischer [4] simply co-opted an estimated carbon content in plumes of 900 ppm from [26], which itself relied on estimates of carbon chiefly in the altered igneous sections of oceanic crust.

Recent work on ocean island basalts has revised the carbon content of their source beneath Hawaii upwards by ∼40% to 263 ppm [27], and the deep mantle associated with the North Atlantic plume beneath Iceland may be much higher (1350 ppm; [23]), although the ultimate source of this carbon remains obscure. The potential long-term contribution of plume material may be estimated from the volume of large, low shear velocity provinces (LLSVPs; [10]), but this does not address the carbon content of these regions, nor the petrological mechanisms of its release. The carbon contents will vary greatly depending on the behaviour of a wide variety of sedimentary rocks during subduction, about which there has been a large number of publications recently, which are outlined in the next section.

**Figure 2.**
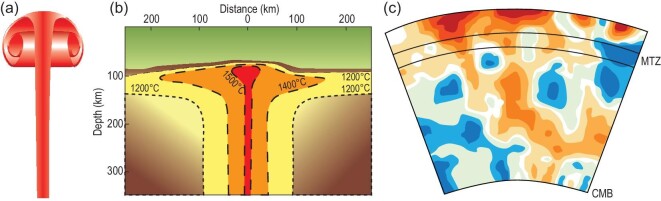
Concepts behind plume inputs into the continental lithosphere. (a) Theoretical and numerical models invoke a narrow plume shaft, usually from the core–mantle boundary, and a large plume head [28,29], which (b) leads to a temperature anomaly of 200–300˚C or more [127]. (c) Since these models were introduced, many tomographic images have been available, which instead show more diffuse tongues of hot material with smaller temperature anomalies (based on [30]), implying that carbon inputs into the lithosphere may need to be revised downwards.

The assessment of plume inputs into the continental lithosphere is also strongly affected by the implicit acceptance and application of a general model for plume dynamics, which is based on numerical models and fluid dynamics, which we refer to as a Campbell-Griffiths plume [28,29]. This is envisaged as a narrow but continuous shaft of hot material, usually emanating from the core–mantle boundary, which spreads into a large plume head up to hundreds of kilometres wide when it encounters the lithosphere (Fig. 2a). However, the many tomographic images of the mantle beneath hotspots, with constantly improving resolution, that have been published since this concept was described do not reproduce this picture. Instead, we see large-scale diffuse tongues of material that are commonly not vertically continuous (Fig. 2b; e.g. [30,31]), implying that carbon inputs into the lithosphere from plumes may be pulsatory and thus laterally and temporally variable.

## CARBON RETURN DURING SUBDUCTION

Although ultramafic rocks undergo carbonation very effectively with low proportions of CO_2_ in mixed CO_2_ + H_2_O fluids [32], such as in seawater alteration settings, the ultramafic section of subducting oceanic lithosphere (peridotite and lower crustal cumulates) probably carries relatively little carbon into subduction zones because very deep fractures are needed for seawater to access the ultramafic layers. This may have been different in periods of Earth's history with abundant slow-spreading ridges, which may have ultramafic rocks at the sea floor as in the modern Gakkel Ridge in the Arctic Ocean [33]. An additional uncertainty for carbon budgets here is the contribution of carbonation of ultramafic rocks along deep faults during flexure of the oceanic crust as it approaches subduction zones [34]. During the last few years, estimates of the carbon content of altered igneous sections of oceanic crust have remained stable (500 ± 100 ppm; [16]), whereas estimates of the CO_2_ flux from the upper sedimentary layers have increased to 57–60 Mt C/yr [35,36]. Nevertheless, the high volume of altered oceanic crust makes this an important carbon reservoir during subduction, whereas sedimentary carbon may be more locally variable.

**Figure 3.**
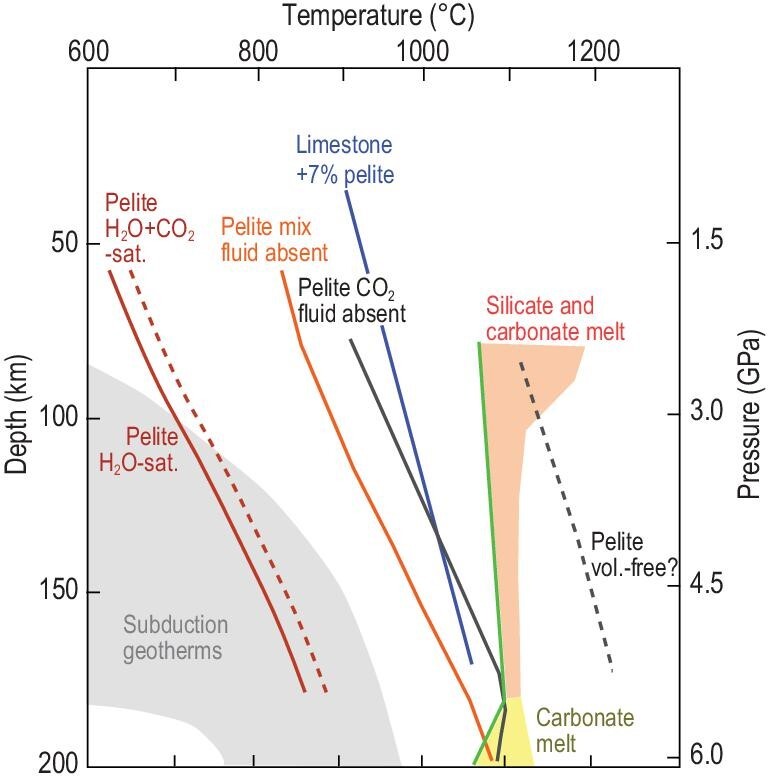
Melting curves for clastic and carbonate sediments compared to subduction geotherms (grey region; [119]). Only fluid-saturated pelitic sediments will melt along modern subduction geotherms. Limestone with a 7% clastic component melts to produce silicate melt [13]; carbonate melts are produced only at high pressures (>5 GPa) from either carbonate or carbonated silicate sediments. Data summarized from [13,37,128].

**Figure 4.**
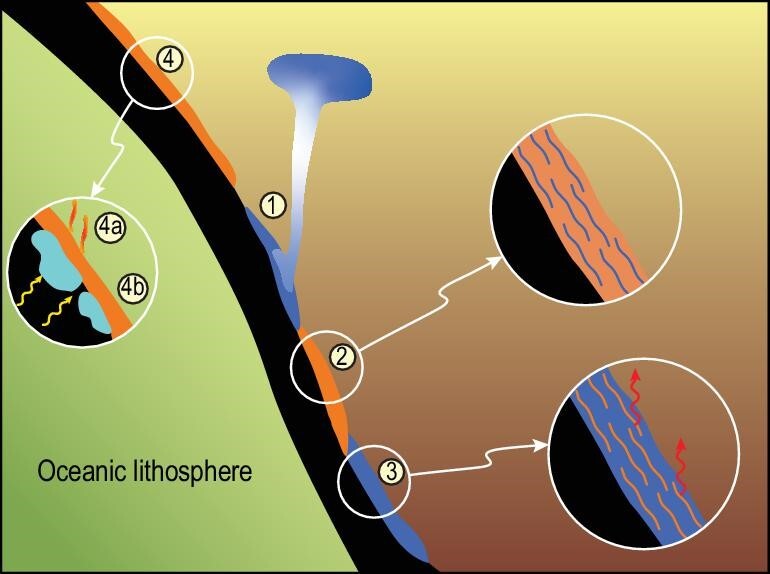
Behaviour of carbonate components in sediments and altered igneous oceanic crust. (1) Thick chalk or limestone sequences will rise diapirically, preventing deep subduction, and become stored in solid form in the mantle wedge beneath arcs [13]. (2) Minor carbonate components in sediments dominated by clastic materials will be subducted unless the clastic sediments rise as diapirs. (3) Predominantly carbonate sediments with minor clastic material may produce minor silicate melts, but not lose their carbon until they stall in the mantle transition zone (Fig. 3). These components and the type of sediment mixture are traceable by Mg and Zn isotopes [52,56,57]. (4) Ophicarbonates in the altered igneous sections of oceanic crust may melt only if fluxed by water from dehydration of the ultramafic rocks below (4a); otherwise they should be deeply subducted if they remain dry (4b) [40].

Perhaps the largest changes that have been seen over the last few years are in our understanding of the behaviour of various types of subducting sedimentary rocks from experimental and numerical modelling work. These include determinations of the melt compositions produced by mixed clastic and carbonate sediments (Fig. 3), as well as the rheological behaviour of, and carbon sequestration in, solid diapirs rising from sedimentary sequences in the subducting slab (Fig. 4). Melts produced by clastic rocks can be summarized as broadly granitic in composition [37], whereas mixed carbonate + clastic rocks result in different melt compositions or may not melt at all, depending on the relative thicknesses of the rock components in the mixture and on the thermal regime of subduction.

Given that the theme of this review is the behaviour of carbon, we restrict our attention to experiments on sedimentary packages with appreciable carbonate content. These have often studied bulk compositions modelled on the average global subducted sediment (GLOSS), which contains carbonate as a minor component (ca. 7 wt%). Recent experiments have shown that the melting behaviour of these differs depending on the amount of water present: when little water is present, phengite remains stable in the dominant silicate part and K-rich carbonate melts appear at 5.5–6 GPa [38]. With excess water, which would correspond to supply from dehydrating serpentinites in the underlying ultramafic rocks of the subducting slab, the melting temperature of the carbonates is strongly depressed (Fig. 3), and dolomite melts out, whereas aragonite persists in the residue and can be subducted to greater depths [39].

However, although GLOSS is widely accepted to be a good estimate of average subducting sediment composition, it is increasingly recognized that local compositional variations of subducting sediments are important [10,11,16]. These variations can substantially affect the subductability of carbonates, meaning whether sediments of various compositions and thicknesses are dispatched deep into the mantle or returned to near-surface conditions as melts or diapirs. At this local to regional scale, a GLOSS composition is not representative of any real rock and so phase relationships of average sediment have little meaning. Nearly pure carbonate sediments such as limestones and chalk form large tracts of sedimentary columns at subduction zones [35], and experiments have shown that their subductability varies with thickness and the thermal regime of subduction. Where carbonate sedimentary sequences are thicker than ∼200 m, they may rise as solid diapirs (Fig. 4) and collect at sublithospheric levels with less than a third proceeding to the deep mantle [13]. Clastic impurities in these carbonate sediments melt first, leading to a small proportion of silicate melt [13], which may assist diapirism by lubrication [12]. Where carbonate sequences are thin and form a minor part of the sedimentary column, they are more likely to be deeply subducted in solid form. Ophicarbonates (carbonated ultramafic rocks) represent another lithology that may be involved in subduction. Here too, melting is promoted by excess water, prompted by the dehydration of serpentinites [40]; only if wet will these form carbonate melts at temperatures appropriate to subduction zones [40,41], in which case widespread deep subduction will be prevented.

Experiments have shown that the dependency of carbonate melting behaviour on temporal changes in the thermal regime of subduction has led to significant changes in the subductability of carbon through geological time [42]. On the modern Earth, carbonates remain stable and are subducted, whereas water is lost as aqueous fluid. However, in warmer regimes that would correspond to Proterozoic or Archean subduction, melting would occur during subduction, initially producing silicate melts at low pressures and then carbonate melts at pressures above ∼4 GPa [42], preventing deep subduction of abundant carbonates. This not only affects the migration and storage mechanisms of carbon as a function of time, but must also mean that the LLSVPs in the deep mantle, which we noted above are thought to be linked to CO_2_ output in plumes, should not be assumed to have compositions corresponding to modern subduction processes.

The subduction of carbon may have been more limited in the distant past before the Phanerozoic ‘explosion’ of skeletal material was available to form thick sedimentary sequences. Stromatolites dominated carbonate sediments early on, but components diversified later [43]. Carbonates also spread in areal extent as oceans become oxic [43], but the prevalence of dolomite in the Proterozoic might have made melting more likely, as noted above [39], counteracting the lower volume of carbonate in the bulk of Proterozoic sediments. There were also temporal fluctuations in the total volume of subducted sediments related to cycles of orogenesis, with times of high sediment load facilitating subduction [44].

However, differences in the thermal regime of subduction are not just a question of age; they may occur in different subduction environments at any one time, although the survival of carbonates and dehydration of slabs is thought to dominate on the modern Earth.

Experiments in the subsolidus regime have shown that the stability and mobility of different carbonates during devolatilization is the opposite of when melting occurs. Here the stability of magnesium carbonates can prevent the removal of carbon, thus favouring its subduction [45,46], whereas it is aragonite (CaCO_3_) that remains stable during melting [40].

In summary, the proportion of carbon that is deeply subducted and proceeds past magmatic processes beneath arcs depends on many factors. It has been estimated as anything between 34%–86% [47] depending on the models used. Once subducted slabs reach the mantle transition zone, however, further stabilization of solid carbonate may be ensured by the transformation to calcium othocarbonate in the solid state (Ca_2_CO_4_) [48,49].

Indications for the deep storage of carbonate at mantle transition zone depths, and its gradual release as it heats up, have received considerable attention in eastern China's ‘big mantle wedge’, which is the region above a stagnant slab in the mantle transition zone [50]. Isotope evidence from nephelinites indicates that here, the carbonate is most probably stored either in former altered oceanic crust (eclogite) or carbonated sediments in the stagnant slab in the mantle transition zone [51–53]. More detailed magnesium and zinc isotopic studies are now beginning to differentiate between the type of sediments involved at source [54–56] and between distinct subducted slabs, as in the case of the Paleo-Pacific and Paleo-Asian oceanic crusts [57]. A similar mechanism has been invoked for other continents, including the Mediterranean and eastern Australia [52,58]. However, the question remains as to how common slab storage in the mantle transition zone has been in Earth history and how effective it is for the long-term carbon cycle. In eastern China, its presence is related to erosion of the cratonic lithosphere, so that the net effect of slab stagnation and craton erosion may lead to a net loss of carbon stored in the lithosphere.

## ENTRAPMENT OF CARBON IN THE CONTINENTAL LITHOSPHERE

Thirty-five years after the recognition that carbonate melts may play a major role as carbon transporters in the mantle [59], it is now established that ‘carbonatitic melts (<15 wt% SiO_2_) are widespread in the Earth's upper mantle and major conveyors of trace and volatile elements’ [60]. Models for the rejuvenation of cratons and the release of the carbon they contain into magmas [61] are backed up by evidence for the release of abundant carbon upon the extensive erosion of the North China Craton lithosphere [62]. Widespread carbonatite metasomatism of the lithosphere is also recorded from younger, non-cratonic areas of the continents such as the Mediterranean and eastern Australia [63,64].

Over the past five years we have gained indications that the amount of carbon in the continental lithosphere from gradual degassing is higher than previously thought, whereas that from plumes may be considerably lower. Plume inputs may be less than from mid-ocean ridges and much less than those from continental rifts [4,65]. There has been a tendency to consider the release of carbon either during the subduction process or in terms of its storage in the continental lithosphere: the deep mantle connection between these two processes needs to be closed in the near future (Fig. 1). This is especially important for the Archean cratonic lithosphere if much of it was formed by the amalgamation of subduction zone mantle wedges or in accretionary orogens [15], because this may incorporate all levels of the supra-subduction capture of carbon, from fluids and melts, through diapirs accumulated beneath arc crust [13], to the stored reaction products of carbonate melts with silicate rocks [66].

Opinions differ as to whether this carbon is stored as carbonate or reduced carbon (graphite/diamond), with the latter more likely in the deep lithosphere due to the general decrease in oxygen fugacity with increasing pressure [67,68]. The definition of cratonic lithosphere has also evolved [69], recognizing that a large proportion of thick lithosphere is younger than Archean (such as below much of Australia [25]). Whilst this may affect models for the formation and stabilization of the lithosphere [14,15] as well as decreasing its average age, it does not appear to greatly change the proportions of cratonic and non-cratonic continental lithosphere [7,4].

### Storage mechanisms

**Figure 5.**
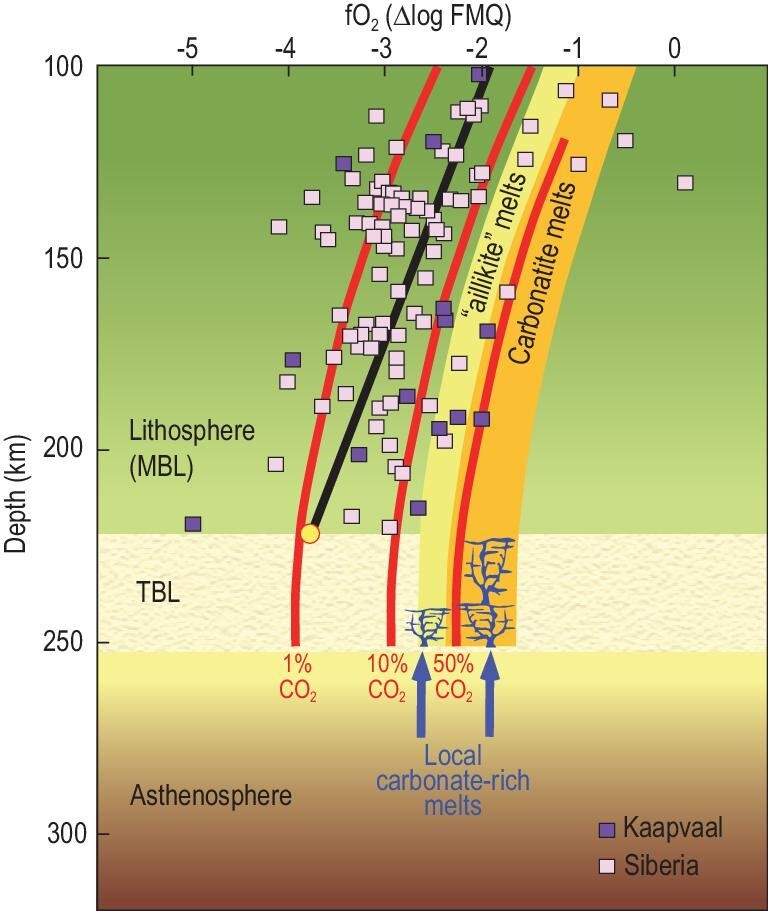
Oxygen barometry of peridotite xenoliths indicates that the cratonic lithosphere is generally too reducing for carbonate-rich melts (xenoliths and red lines marking carbonate contents of melts from [67]; solid straight line shows average). Episodic local carbonate-rich melts from just below the lithosphere or from subducted material (arrows) cause interaction between oxidized melt and reduced rocks (circle), resulting in diamond precipitation or local carbonate-phlogopite veins and dykes [61,101]. MBL = mechanical boundary layer; TBL = thermal boundary layer.

**Figure 6.**
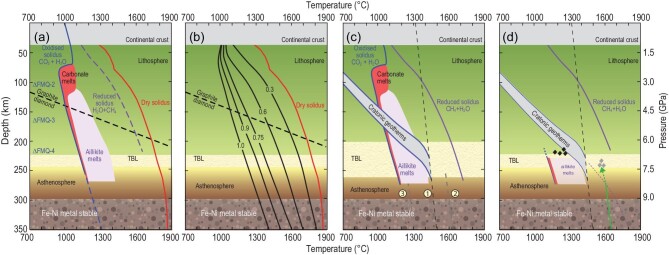
Melting curves of mantle peridotite in oxidized and reduced conditions and their relationship to likely geotherms. (a) Possible melting curves in the lithosphere: the oxidized solidus for peridotite with CO_2_ and H_2_O [71], has shaded areas for melt compositions (carbonatite and aillikite [10%–30% SiO_2_]). The dashed line for reduced solidus is the most appropriate solidus, corresponding to reducing conditions with low water activity [68,129]. (b) Melting curves for reduced conditions; curves labelled with water activity in H_2_O + CH_4_ mixtures [129]. These curves are relevant for the asthenosphere, where conditions are mostly too reduced for the oxidized solidus in (a) to apply [98]. (c) Cratonic geotherms fit to an isentrope of 1315˚C (dotted line (1) [7,72]), showing that melts would be widespread in the lithosphere if conditions were oxidized, but they are not: the reduced solidus is not reached. Dotted curves (2) and (3) apply to upwelling hot material and regional downwelling, respectively, emphasizing that curve (1) is just a global average. The TBL is wider here to account for the full range of geotherms shown. (d) Summary of the most likely real-world melting curves for variation of the oxidation state with depth. No melting in the lithosphere gives way to two types of melts in the upper asthenosphere and TBL: oxidized, carbon-rich melts as in (a) are restricted to the uppermost asthenosphere and mostly solidify to deposit diamond (dark diamonds). Reduced upwelling mantle from the deeper asthenosphere will melt when it meets the solidus (dotted line) and these melts solidify on encountering the more reduced TBL/lithosphere, depositing polycrystalline diamonds (light diamonds). These melts are more oxidized than their source due to dissolution of volatiles in the melt.

The storage and re-release of carbon in the lower lithosphere depends on pressure, temperature and oxygen fugacity as well as on reactions that occur between infiltrating melts and silicate mantle rocks. We consider these factors now in more detail, emphasizing that local variations in redox, pressure, temperature conditions, and rock and melt compositions, will lead to the coexistence of rock and melt types that are usually considered to be mutually exclusive in global models (Figs 5, 6).

Carbon is thought to be transported as carbonate-rich melts, but deposited in reduced form as diamond at depths greater than ∼140 km by redox freezing [4,67,70]. The oxygen fugacity of mantle rocks decreases consistently with increasing pressure and lies between ΔFMQ −2 to −4 for most cratonic xenoliths, which is too reducing to coexist with melts with any appreciable amount of CO_2_ (Fig. 5). In Fig. 5, the shaded areas for carbonate-rich melts (from [71]; Fig. 6a) are guided by the calculated CO_2_ contents in melts (coloured lines; [67]). The average fO_2_ of the lithosphere (black line in Fig. 5) is more reducing than the convecting mantle below it, which in turn depends on the depth of metal saturation in the Earth's mantle [70]. The lithosphere owes its more reduced state to substantial melting in the Archean, which fractionated its oxidation state, leaving a more reduced residue [68].

### The petrological nature of the thermal boundary layer

The lithosphere–asthenosphere boundary is not a sharp boundary but a transition—the thermal boundary layer (TBL; beige zone in Fig. 5)—which may be between 20 and 40 km thick [72,7,73], depending on surface heat flow and the local temperature in the underlying asthenosphere. Any carbonate-rich melts infiltrating from below are more oxidized than the lithosphere and will freeze somewhere in the TBL as they encounter more reducing ambient conditions, forming either diamond [66,70] or carbonate-rich veins if the redox reaction does not proceed to completion (Fig. 5).

High-pressure experiments have outlined the melting curves of peridotite in the presence of different volatiles. In the lower cratonic lithosphere, peridotites generally contain little water or carbon [74,75] and conditions are reducing, so that the solidus lies at much higher temperatures than the geotherm (Fig. 6b; dashed purple line); this position of the solidus applies for low water activity (Fig. 6b).

In the deeper mantle, the oxidized solidus (blue line in Fig. 6a [71]) is only relevant for the uppermost asthenosphere because the fO_2_ deeper in the mantle is too low to support carbonated melts. It may also apply locally in the TBL and lithosphere where carbonate-bearing rocks have formed from the infiltration and solidification of oxidized melts (Fig. 5). Otherwise, melting will not occur in the TBL or lithosphere because the reduced solidus is too high (Fig. 6b). Although the TBL can be seen at a global scale as a zone of transition between the more reduced lithosphere and more oxidized upper asthenosphere, the rock types and melts within it will vary greatly in fO_2_ on a local scale. This means that melts may occur locally, whereas other parts of the mantle at essentially the same depth remain solid and reduced. There is no such thing as ‘*the* oxidation state of the mantle’, particularly at this critical zone at the base of the lithosphere.

Next, we note that the position of the cratonic lithosphere geotherm and the depth of the transition into the TBL in most illustrations also depend on a global average, namely the 1315˚C isentrope for the asthenosphere, which is chosen to account for the average oceanic crust thickness [72,7]. This is a steady state geotherm, whereas the formation and re-melting of carbon-rich assemblages are episodes of perturbation. Variations are therefore depicted in Fig. 6c, illustrated by both upwelling of mantle (‘plume’) as well as regional downwelling that will periodically occur. It can be seen from Fig. 6c that if oxidized conditions prevail, the geotherm usually lies well above the solidus: melts here will therefore be aillikite in composition ([71]; ca. 20 wt% SiO_2_) and not the carbonatites (2–5 wt% SiO_2_) that would appear at the solidus. To achieve carbonatites at this depth would require cooling, which is now thought to be the case for carbonatites of the Toro Ankole volcanic field in Uganda, which lies in a region of major downward current in mantle convection [76]. In summary, in the TBL and lower lithosphere, there may be fairly major melting in oxidized regions (20%–30%), whereas most of the mantle at this depth is reduced and so does not melt at all.

In conclusion, the TBL is a petrologically heterogeneous zone consisting of a mixture of rock types and oxidation states in which melts probably move through ephemeral veins and dykes [77]. The presence of these small melt pockets weakens the TBL, in places increasing its vertical extent, as shown in Fig. 6.

### Composition and effects of melts infiltrating from the asthenosphere

At the top of the asthenosphere, the fO_2_ is initially higher than in the reduced, depleted lithosphere, but decreases with increasing depth until metal saturation is reached somewhere between 250 and 350 km [70]. The dotted purple curve in Fig. 6d shows a preferred solidus for the range of redox conditions towards more reduced conditions at greater depth; this curve moves to significantly lower temperatures as it approaches the TBL because of the increasing *a*H_2_O (i.e. increasing H_2_O/CH_4_) with increasing fO_2_; the high temperature of the solidus at >280 km is caused by low *a*H_2_O, which is partly due to the high activity coefficient of CH_4_ [78]. The exact fO_2_ profile and the position of the reduced solidus are only approximately known: if conditions are more reducing than in Fig. 6d, metal saturation will occur at shallower depths and melting will begin closer to the base of the lithosphere.

If we assume the reduced solidus in Fig. 6d applies at depth, mantle upwelling along the green line will melt at ∼270 km, with the exact depth depending on the geotherm and *a*H_2_O. High-pressure experiments in reducing conditions (i.e. with H_2_O + CH_4_ instead of H_2_O + CO_2_) show that melts are Mg-richer than under oxidized conditions at the same pressure, containing 20%–35% MgO [79,80]. Melts produced in this depth window in reducing conditions are extremely magnesian because of the incongruent production of orthopyroxene and preferential melting of olivine [81]. MgO-rich melts are usually assumed to be produced by extensive melting, as in the case of komatiites, but could represent much lower degrees of melting when generated at these pressures. Because most of the upper mantle is in a reduced state, these melts will normally reach the TBL and the base of the lithosphere without encountering any oxidized assemblages containing carbonate. Here, they will interact with peridotite at fO_2_ too low for carbonate stability, and will transform the peridotite towards websterite; this process has been used to explain the increase in modal orthopyroxene in cratons such as Kaapvaal [81]. Evidence for this process is preserved in the compositions of olivines at Jericho (Slave craton), which can be modelled by crystallization from ‘komatiitic’ melts at greater depths than oxidized metasomatism can occur [82]. Interaction with these MgO-rich melts consumes olivine and produces orthopyroxene from the incongruent melting of clinopyroxene [81], which also accounts for the absence of websteritic clinopyroxene. Wehrlitic garnets would result if the reaction had occurred in more oxidized conditions with carbonated melts [83], but are absent in the polycrystalline diamond inclusion suite.

This melt/rock interaction also results in the deposition of polycrystalline diamond aggregates, which are rocks consisting *mostly* of diamond, in which silicate minerals are merely interstitial accessory phases [84]. We propose that these may be a main reservoir of ‘redox frozen’ carbon in the lower lithosphere and particularly the TBL. The deposition reaction explains why the mineral suite of polycrystalline diamonds contrasts with most diamonds in being dominated by websteritic garnets (57% vs. 3% in peridotitic diamonds), whereas clinopyroxenes have compositions typical of peridotites and olivines are notably absent [84]. The carbon for the diamonds may be supplied by the reduced melts, in which carbon solubility is low but not insignificant [85], or from locally pre-existing carbonates. Some micro-inclusions in polycrystalline diamonds indicate strongly reducing conditions, and an association of websterite with metal and cohenite formed by the reduction of local carbonate [86,87].

### Summary of carbon storage mechanisms in the TBL and lithosphere

**Figure 7.**
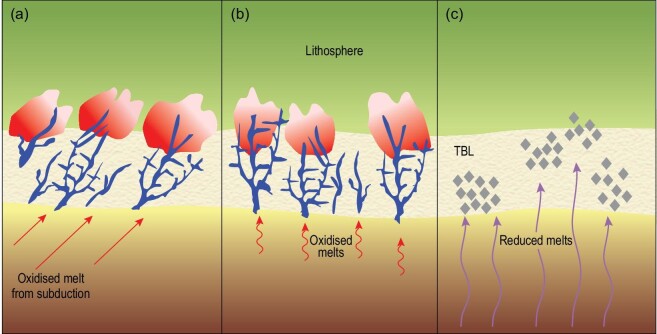
Precipitation mechanisms for carbon in the TBL and lower continental lithosphere. (a) Oxidized, carbonate-rich melts (red arrows) may originate from subducted materials. These initially solidify to form diamonds by redox freezing [70,61]. Both diamond and carbonate may exist in close proximity due to local variations in oxygen fugacity and melt flux. (b) Oxidized, carbonate-rich melts may be produced from peridotite [71,130] or other ultramafic rocks in oxidized patches of the uppermost asthenosphere. At higher influxes of melt, carbonates will be deposited (dark vein system; the carbonation freezing front [66]). Reactions between carbonate-rich melts and peridotite produce a zone with minerals characteristic of hydrous mantle metasomatism [99,101]. (c) Reduced melts emanating from deeper in the asthenosphere (Fig. 6d) never attain the oxidation state necessary to form carbonate, depositing diamonds as polycrystalline diamond aggregates associated with websteritic silicate minerals [84,86]. These may be easily remobilized when overprinted by carbonate melts as in (a) and (b).

Figure 7 summarizes the carbon inputs into the continental lithosphere, focussing on the mechanisms rather than the abundances. The first is incorporation during lateral accretion of lithosphere (Fig. 7a), which may apply to the formation of continental lithosphere where it originates by assembly during accretionary orogenesis. This may include large tracts of solid, unmelted carbonate that have risen diapirically from subducting slabs and become stored beneath arcs [13] and later incorporated into the continental lithosphere, especially in periods of the Phanerozoic when carbonate platforms were abundant [88,89]. Carbonate-rich melts may originate during subduction, either when carbonate is a minor component in clastic sediments [38,39] or from melting of altered oceanic crust [66]. In the latter case melting is likely at >300 km because of a major backbend in the solidus [90], which may help to stabilize stagnant slabs. Thinner sequences of limestone and chalk will remain solid and be deeply subducted along most modern subduction geotherms [13], but earlier in Earth history they may have melted and so contributed significantly to lithospheric carbon reservoirs along warm or hot geotherms [42].

A direct link between subducted carbonate and the storage of carbon in the cratonic lithosphere has become apparent recently from experiments that find solid salts in reaction zones between melts of subducted sediments and peridotite [91], or coexisting with carbonate melts along warm subduction geotherms [42]. These salts occur only at pressures of 4 GPa and above, and are intermediate in composition between NaCl and KCl and mimic the compositions of fluid inclusions in diamonds [92]. A similar association of alkali chlorides and carbonates is increasingly invoked for the source regions of kimberlites, which are quintessentially cratonic in occurrence. Originally described from exceptionally fresh kimberlites at Udachnaya (Siberia) [93], this chloride-carbonate suite has been identified in kimberlites from other cratons [94] as well as in sheared peridotites [95] and in the megacryst suite, which is thought to originate from magmatic events that do not escape the deep cratonic mantle [96].

Regardless of the origin of the carbonate introduced into the lithosphere, its storage usually occurs in reduced form as diamond or graphite by a process variously labelled as redox freezing [70,86,4] or a carbonation freezing front [97], given the conclusion that ‘an oxidized mantle … is the exception rather than the rule’ [98]. Experiments have demonstrated this freezing process and also show that it results in products reminiscent of assemblages formed during mantle metasomatism (Fig. 7b; [99,100]). The storage of carbon in reduced form that is later released as CO_2_ is further supported by low W/U and W/Th ratios in magmas, since W^4+^ in reduced conditions is retained in the residue during melting [63]. In areas of the TBL and lower lithosphere where the fO_2_ is slightly higher or a large amount of carbonate melt is introduced, phlogopite- and carbonate-rich metasomatic vein networks should result. This process has been experimentally demonstrated [101], and similar assemblages have been invoked as essential source components of alkaline igneous rocks, providing a link between the long-term storage of carbon and potassic magmatism [102,103].

An additional source of diamond in the lower cratonic lithosphere unrelated to subduction is melting deeper in the mantle in reduced environments (Figs 6b, d, 7c). This may result in considerable diamond deposition for later reactivation, stored as polycrystalline diamonds: these are generally thought to be rare, but may represent up to 30% of diamond production at a single kimberlite pipe [84]. They are probably concentrated at the deepest lithospheric levels (Fig. 6b) and so have low survival potential once they are remobilized and encounter a more oxidized environment.

Options (and mechanisms) for carbon incorporation into the continental lithosphere have multiplied in recent years, but it is currently very difficult to assess the amount of carbon stored by each of these mechanisms. This will become clearer with further work on the melting and rheological behaviour of different rocks during the subduction process and further reaction experiments, particularly those studying the sedimentary components of subducted slabs. A variety of melts, both oxidized and reduced, lead to the storage of carbon in the lithosphere, whereas carbonate-rich melts are the main agents that eventually release this carbon to the surface [104].

## CONTINENTAL RIFTS AND CRATON EDGES—REJUVENATION OF LITHOSPHERE AND CARBON RELEASE

The release of carbon from the continental lithosphere takes place from volcanoes and diffuse degassing along faults [4,6], primarily associated with rifting. There is a well-known spatial association of strongly carbonate-rich magmatism with the edges of cratons, exemplified by the East African Rift, where the carbonate content of igneous rocks increases as the rift approaches the craton [4,105–107]. Worldwide, it is estimated that 75% of carbonatites occur within 600 km of craton edges [108]. More recently, a similar spatial association between craton edges and metal deposits has been delineated; 85% of giant sediment-hosted base metal deposits occurring within 200 km of a major step in lithosphere thickness [109]. This raises the question of whether there is a genetic link between the carbonate content of mantle melts and the transport of metals, which is a topic of current research. High platinum group elements (PGE) contents in nephelinites and basanites in eastern China may be caused by oxidative breakdown of sulphides and the transport of PGEs in carbonate melts [110]. On a smaller scale, coupled transmission electron microscopy and Laser-ICP-MS investigations of experimental carbonate melts indicate that Ni, Co, Cu and PGE may be scavenged by carbonate melts [111] and thus remobilized when melting occurs at the continents’ base.

**Figure 8.**
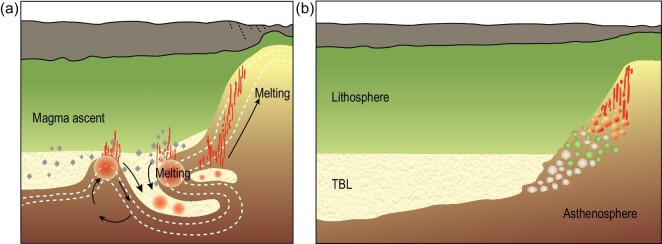
Competing proposed mechanisms for the erosion of cratonic roots. (a) *Edge-driven convection* is concentrated at step-changes in lithosphere thickness [112,114]: movement in the convecting mantle tears rheologically weak sections of the thermal boundary layer (also known as the rheological boundary layer [131]) and the lowermost lithosphere downwards and sideways. This results in the heating and oxidation of diamond causing the production of carbonate-rich melts, which migrate upwards primarily at the edge of cratons. Sketch partly after [73]. (b) *Lateral advection* of crumbled solid pieces of the TBL and lower lithosphere (after [107]) is promoted beneath developing rifts. Melts are produced by oxidation of reduced carbon, heating and decompression, explaining the concentration of carbonate-rich melts at the juncture of cratons and rifts [4].

Early models for the rifting of cratonic lithosphere to form carbonate-rich magmatism at their edges assumed the action of thermal plumes was necessary [61], but recent work has put forward two competing mechanisms. The first is edge-driven convection, in which lateral movement of the convecting mantle strips the lowermost layers of the craton (Fig. 8a, [112,73]). Small-scale convection has long been thought to occur in the TBL [113], but edge-driven convection at the craton margins is more effective due to a step-change in lithosphere thickness [112]. It is now confirmed as a possible mechanism by 3D geodynamic models and applied to widespread volcanism at the edge of the eastern Australian craton [114]. Gernon *et al.* [73] considered the smaller-scale petrological implications in which the TBL and lower lithosphere are ‘torn down’ into the asthenosphere (Fig. 8a) and applied this to the origin of kimberlites. This process assists heating and oxidation by re-immersing rocks of the TBL in the upper asthenosphere, thus optimizing the production of carbonate-rich melts from stored reduced carbon (diamond). This means that with the transformation of diamond to carbonate, little evidence is likely to survive for the previous existence or origin of diamond, nor for the potential of this section of the mantle for diamonds. Redox melting is likely to be very important in the origin of the carbonate-craton edge association [61,62,104].

The second mechanism appeals to lateral advection that causes a crumbling of the solid lower lithosphere [107], moving it 100 km or more to the craton edge where it will rise beneath the developing rift and melt due to the heat from the surrounding asthenosphere coupled with decompression (Fig. 8b). In both mechanisms, the melting of volatile-rich hydrous pyroxenite assemblages with universally lower melting points than peridotite [115] promotes the production of H_2_O and CO_2_-rich melts. Also, these erosive forces will concentrate carbonate-rich material towards the edges of the cratons as melts will follow the topography of the underside of the lithosphere from the centre towards the edges [116].

## LOCAL VERSUS GLOBAL, AND CHANGES THROUGH GEOLOGICAL TIME

A central theme of this review is to emphasize the effects of local differences in mantle source rock type, thermal conditions and redox state on the deposition and remobilization of carbon in the continental lithosphere. The importance of spatial variations is increasingly recognized and studied [10,11,16,42]. Whilst the overall goal is often to assess global carbon budgets, the use of global average compositions can lead to misleading conclusions, or hide important differences in the melting reactions of specific rock types. Carbon is transported by melts, the compositions of which depend on the mineral assemblages in the source and on melting reactions, not on average geochemical compositions. The use of average compositions may be justified for voluminous melt types that are the collected products from large source volumes, such as mid-ocean ridge basalts, and to a lesser extent ocean island basalts and continental flood basalts, but the deposition of carbon and the generation of carbonate melts relevant to the deep carbon cycle are local, low-volume phenomena. This enables, for example, phlogopite-carbonate dykes to exist close to the base of the lithosphere [101,102] in a zone that is mostly characterized by reduced conditions where carbon is present in elemental form as diamond (Fig. 6d).

Other volatiles and incompatible components are equally likely to be concentrated into distinct rock types, such as H_2_O and many incompatible trace elements in hydrous pyroxenites. Since the hydrous minerals melt first [115], the melts produced are mostly determined by the compositions of these minerals, corresponding to melilitite to lamproite, and differing from the basaltic melts typical of peridotite sources [117]. Lamproite source regions are often reduced and so may contain carbon as diamond [118], and carbonates in hydrous pyroxenites, where present, are not constrained to be dolomite or magnesite as in peridotites. The stability and melting behaviour of specific carbonate minerals may differ widely [45,46].

The subduction system is replete with variations: subduction geotherms vary at any one time [119], as do the amount and composition of sediments in the subduction channel (Fig. 3). Whether carbonate in sediments melts or not depends on the thickness of the sediments on the downgoing slab as well as on the geotherm [10,42,88]: diapirism may prevent deep subduction for thick limestones and chalk, as well as carbon caught up in predominantly clastic sedimentary packages [120]. The oxygen fugacity at which reactions occur plays a role here too, because carbon in the reduced state (organic carbon) may be preferentially retained in the downgoing slab, leading to its deep subduction [121].

The carbon budget in the continental lithosphere is also affected by the time-integrated effects of carbon deposition and remobilization, which depend on mechanisms that may also have changed over geological time. These are beyond the scope of this review, but a few comments will give pointers to future work that will be needed to clarify carbon inputs into, and outputs from, the lithospheric reservoir.

Just as subduction geotherms vary greatly on the modern Earth [119], they will have varied in the past, but may have been disproportionately hotter if greater ocean ridge length and smaller plates were the solution to facilitating greater heat loss on a hotter Archean Earth [122]. This hotter subduction would have enabled melting of the oceanic crust, explaining the large peak in tonalitic continental crust formation in the period 3.0–2.5 Ga. This has large effects on carbon behaviour as well, as carbonate sediments will melt along hotter subduction geotherms [42], throttling the subduction of carbon. The oxidation state of the mantle may have been lower at these times [123], in which case hot subduction may further favour carbon transport into, and its capture in, the mantle wedge [100]. The diapirism of both carbonate and clastic sediments (Fig. 4) probably did not occur, with carbon transported by melting instead. The big mantle wedge and stagnant slab beneath China (Figs 1 and 7) are thought to provide carbon components in the form of carbonate melt components on the modern Earth [52,57], but it is not clear how typical such stagnant slabs have been in Earth history.

Our understanding of carbon subduction and return is often subject to generalizations by the use of averages and archetypical examples of geodynamic and petrological processes, and we should beware of oversimplifications in seeking to fine-tune budgets in the carbon cycle. Figure 2 draws attention to the widespread application of theoretical concepts of the size, intensity and shape of mantle plumes. The Hawaiian plume is often taken as the archetypical plume and yet is the exception on the modern Earth. The same is true of the East African Rift for continental rifts, and the ‘Big Mantle Wedge’ and stagnant slab in East Asia; how typical are these as we go back in Earth history? If accretionary orogens were more common before the growth of large continents, then the existence of deep subduction into the mantle transition zone and lower mantle could be doubted, which would greatly affect carbon recycling. The GLOSS average global subducted sediment has been used in high-pressure experiments, even though this composition does not exist in realistic, small-scale scenarios: it is the individual rock types that need to be studied. Another crude average of relevance to the processes outlined in this review is *the* oxidation state of the mantle, an estimated average value that contrasts with the local variations depicted in Figs 5–7. A third example is the cooling curve of the mantle, which is usually presented as a single cooling curve for the whole mantle, whereas regional variations are relevant for the development of the deep carbon cycle through time.

Significant advances in our understanding in the near future will most likely come from combinations of isotope systems such as those currently used to recognize different carbonate-bearing rocks in the sources of volcanic rocks [54–58]. High-pressure experimental studies in the last few years have shown the value of reaction experiments, in which more than one rock type is included in the same experiment. These have produced unexpected results that were not found in studies of single rocks, such as the occurrence of salts in reaction zones [42,91], which are similar to those found as inclusions in diamonds and kimberlites [92,94]. Further studies at higher pressures will help to forge the link between input into the mantle at subduction zones and flux from the mantle into the continental lithosphere. Integration of petrological and geophysical work will modify interpretations of geophysical signals to focus on minor rock types rather than seeking explanations in terms of average mantle rocks. Examples are electrical conductivities that are possibly linked to phlogopite pyroxenites [124], garnet pyroxenites [125] or reactions between carbonate melts and silicate rocks [126].
